# Case report: Hemophagocytic lymphohistiocytosis in a child with primary immunodeficiency infected with *Talaromyces marneffei*

**DOI:** 10.3389/fimmu.2022.1038354

**Published:** 2022-12-02

**Authors:** Huimin Yan, Yunjun Mo, Shilin Liu, Xiaojuan Luo, Lianlian Liu, Lintao Zhou, Xiuming Zhang, Yunsheng Chen, Ke Cao

**Affiliations:** ^1^ Clinical Laboratory, Shenzhen Children’s Hospital, Shenzhen, Guangdong, China; ^2^ Medical Laboratory, Shenzhen Luohu People’s Hospital, Shenzhen, Guangdong, China; ^3^ Division of Hematology and Oncology, Shenzhen Children’s Hospital, Shenzhen, Guangdong, China

**Keywords:** hemophagocytic lymphohistiocytosis, *Talaromyces marneffei*, immunodeficiency disease, *CD40LG*, *CARD9*

## Abstract

Hemophagocytic lymphohistiocytosis (HLH) is a life-threatening immune-mediated disease that affects patients with known genetic defects and is increasingly found among those with autoimmune diseases and persistent infections. *Talaromyces marneffei* (TM) is a human opportunistic fungus that commonly infects immunodeficient or immunosuppressed individuals. Few TM-associated secondary HLH cases resulting from autoimmune deficiency have been reported previously. The current case study describes a pediatric patient hospitalized with recurrent fever and lymphadenopathy. The child had abnormal blood cell classification, and microscopy revealed mature granulocytes that phagocytized fungal spores. It was speculated that the patient was infected with TM. The pathogen was detected earlier than the blood culture and confirmed by metagenomic next-generation sequencing. Whole-exome sequencing revealed that the patient had complex mutations associated with immunodeficiency. This included a mutation in exon 3 of the *CD40LG* gene, c.346G>A, which may be linked to hyper-IgM syndrome, a primary immunodeficiency disease with immunoglobulin conversion recombination defects that could explain the patient’s increased susceptibility to serious opportunistic infections. In addition, a heterozygous frameshift variant, c.820dup (p.Asp274GlyfsTer61), was detected in exon 6 of *CARD9*, a key gene associated with fungal immune surveillance. After 4 days of fungal treatment, the abnormal blood cell clusters disappeared, but other infections occurred in succession for 6 months after rehabilitation. The patient was followed with the aim of providing subsequent immunotherapy. This study found that infection can trigger HLH in HIV-negative individuals, highlighting the importance of early definitive identification of the causative agent and investigation of potential immunodeficiency.

## Introduction

Hemophagocytic lymphohistiocytosis (HLH) is a type of histiocytosis that is clinically characterized by persistent fever, hepatosplenomegaly, pancytopenia, and hemophagocytosis in the bone marrow, spleen, or lymph nodes (1). HLH is primarily divided into two classes: primary and secondary. While primary HLH is caused by defined genetic mutations, secondary HLH is thought to occur in response to infections, tumors, or autoimmune diseases. HLH is one of the complications of Epstein–Barr virus (EBV) infection (2), and a multicenter study found that bacteria and fungi can also trigger HLH (3). *Talaromyces marneffei* (TM) is a rare opportunistic pathogenic fungus that can cause fatal disseminated mycoses in immunocompromised hosts. Bamboo rats are the natural hosts for TM, and both animal–animal and animal–human transmission, respectively, can occur (4).

Healthy immunocompetent individuals are usually able to clear TM, while immunocompromised people are at risk of developing an active or latent infection that can be reactivated later. TM infection occurs predominantly in HIV-infected individuals, and TM infection also occurs in HIV-negative patients, most of whom have congenital immunodeficiencies (5). A number of genetic disorders are associated with congenital immunodeficiency, including mutations in *CD40LG* and *CARD9*. *CD40LG* encodes CD40 ligand (CD40L), a member of the TNF superfamily that is primarily expressed on the surface of activated T lymphocytes, where it binds to CD40 and plays an important role in B lymphocyte proliferation, germinal center formation, and immunoglobulin class switching (6). *CD40LG* deficiency is the most common cause of hyper-IgM syndrome (HIGM) which has an X-linked recessive inheritance pattern. HIGM is associated with defective immunoglobulin switching recombination, resulting in lower IgG, IgA, and IgE levels and normal or elevated IgM levels, defects that increase a patient’s susceptibility to recurrent and life-threatening opportunistic infections (7, 8). CARD9, a key adaptor molecule involved in C-type lectin receptor signaling, is primarily expressed on myeloid cells and plays an important role in anti-fungal immune response. *CARD9* deficiency is associated with phaeohyphomycosis, an autosomal recessive inheritance pattern (9). The current report describes a case of secondary HLH in a child with a *CD40LG* mutation that may be linked to an increase in TM infections. A coexisting *CARD9* mutation was also identified and discussed. This study enhances our clinical understanding of the role of *CD40LG* and *CARD9* mutations in increasing susceptibility to fungal infections.

## Case description

A 6-year-old boy was admitted to our hospital with a 3-week history of fever and neck mass. He was initially seen in the local infectious diseases hospital after a chest radiography indicated potential acute milia pulmonary tuberculosis; however, the cause of the fever remained unclear after subsequent examinations. After showing minimal response to antibiotic treatment, he was transferred to our hospital. Upon admission, a physical examination revealed scattered bleeding spots on the extremities, swollen eyelids and lips, and thrush in the oral cavity, but no rash, joint swelling, or joint pain. The breathing sounds were slightly coarse in both lungs, and moist rales were evident, but the heart sounds were normal. The patient’s abdomen was distended, and a neurological examination showed no significant abnormality. The chest computed tomography (CT) examination revealed enlarged mediastinal lymph nodes, and the abdominal CT showed significant splenomegaly, multiple mesenteric and retroperitoneal lymphadenopathy, and a small amount of pelvic effusion. Gastroenteroscopy revealed multiple ulcers in the rectum and sigmoid colon.

The serum high-sensitivity C-reactive protein level was 88.25 mg/L (reference range: <10 mg/L), and the procalcitonin level was 6.65 ng/ml (reference range: <0.05 ng/ml). The routine full blood count results revealed pancytopenia with a white blood cell count of 1.38 × 10^9^/L (reference range: 4.3–11.3 × 10^9^ cells/L), a hemoglobin level of 86 g/L (reference range: 110–160 g/L), and a platelet count of 86 × 10^9^/L (reference range: 100–300 × 10^9^ cells/L). In addition, the serum ferritin level was 87,741.30 ng/ml (reference range: 22–322 ng/ml), the fibrinogen level was 1.82 g/L (reference range: 2–4 g/L), and the triglyceride level was 2.69 mmol/L (reference range: <1.7 mmol/L). The cellular immune function tests indicated that 52.29% of the cells were CD3+, 27.32% were CD3+CD4+, 23.47% were CD3+CD8+, 41.85% were CD19+, and 2.07% were CD16/56+. B lymphocyte subsets were also detected, of which 95.30% were naïve B cells, 0.50% were memory B cells, 3.90% were transitional B cells, and 0.20% were plasmablasts. The immunoglobulin profile revealed an IgG level of 3.85 g/L (reference range: 5.28–21.90 g/L), an IgM level of 1.80 g/L (reference range: 0.48–2.26 g/L), and an IgA level of 0.33 g/L (reference range: 0.41–2.97 g/L). Additional results are found in **Supplementary Table S1**. The patient’s HIV serology was negative. The bone marrow smears revealed the presence of hemophagocytosis and fungal spores. In summary, the child had a fever for >7 days, followed by generalized lymphadenopathy, pancytopenia, decreased fibrinogen levels, significantly elevated triglycerides and serum ferritin levels, and an enlarged spleen. According to the HRH-2004 guidelines, the child could be diagnosed with HLH (10).

**Figure 1:**
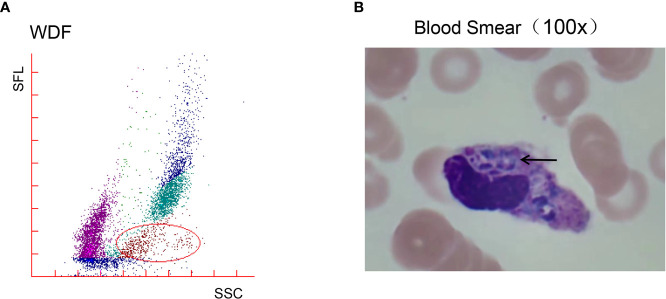
White blood cell (WBC) count analysis revealed abnormal cell masses and a microscopic morphology. **(A)** The WBC differential channel of the Sysmex XN-3000™ Automated Hematology Analyzer suggests the presence of abnormal cells. The area shown in the circle originally belonged to mature granulocytes, but the scatters were not concentrated, and the apparatus defined these cells as eosinophils (shown in red). **(B)** A peripheral blood smear revealed scattered phagocytic phenomena in fungal spores and mature granulocytes, with oval fungal spores, small nuclei, a purple color, deviation to one side, pale blue plasma, and the entire profile resembling corn, with a morphology consistent with *Talaromyces marneffei*.

**Figure 2:**
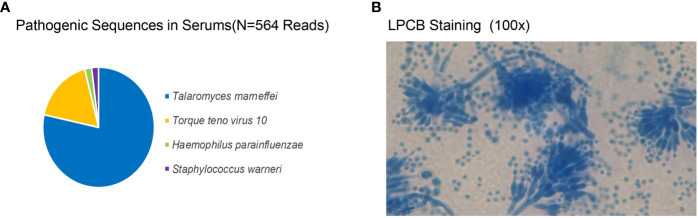
*Talaromyces marneffei* identified by metagenomic next-generation sequencing and Sanger sequencing. **(A)** The pathogen sequences (*N* = 564 reads) identified in the patient’s plasma included *Talaromyces marneffei* (*N* = 539; 86.80%), *Torque teno virus 10*, *Haemophilus parainfluenzae*, and *Staphylococcus warneri*. **(B)** After staining the blood culture isolate at 25°C with lactophenol cotton blue, the morphology of branching septate hyphae and typical brushlike cladings could be observed. The isolate was sequenced by Sanger, and the genbank number was OP744583.

**Figure 3:**
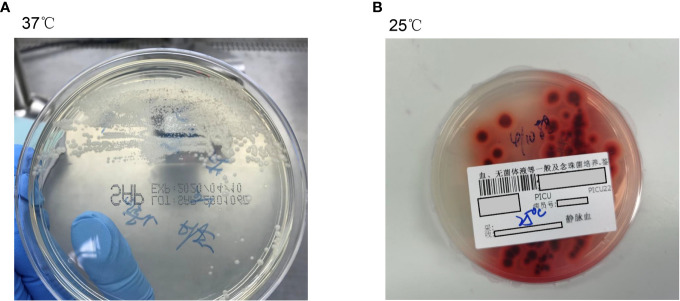
The peripheral blood culture revealed an infection with *Talaromyces marneffei*. **(A)** Peripheral blood culture-positive samples were inoculated in Sabouraud medium at 37°C, and the colonies were bacterial-like, glabrous, colorless, initially dull, and later gyral, without diffused pigments. **(B)** At 25°C, *Talaromyces marneffei* grew rapidly, with broom-like branches and spore chains unique to *Penicillium*, indicating a hyphal phase. The characteristic water-soluble wine-red pigment was produced after 2 to 3 days, staining the medium or colonies red.

The initial blood cell analysis suggested the presence of abnormal cells, which were identified as mature granulocytes engulfing fungal spores (**Figure 1**). After consulting the medical records, it was suspected that the patient had TM infection. Metagenomic next-generation sequencing (mNGS) and Sanger sequencing were used to identify infectious pathogens for further confirmation (**Figure 2**). The subsequent mNGS of the bronchoalveolar lavage fluid and blood culture supported TM infection (**Figure 3**). There was no evidence of autoantibodies, and bone marrow and lymph node biopsies and CD40 expression excluded autoimmunity and a possible hematologic neoplasm. The cellular immune function tests revealed a decrease in the number and activity of NK cells, lower CD107a expression, normal levels of hemophagocytic proteins—such as perforin, granzyme B, SAP, and XIAP—and an sCD25 expression level of 7,630 U/ml (>2,400 U/ml).

**Figure 4:**
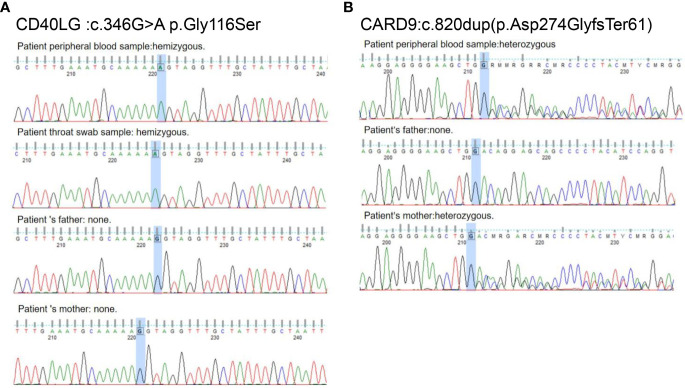
Family investigation of *CD40LG* and *CARD9*. **(A)** c.346G > A p.Gly116Ser in the *CD40LG* exon 3 is an acquired novel variant in this patient. **(B)** c.820dup (p.Asp274GlyfsTer61) in *CARD9* exon 6 was inherited from the patient ‘s mother.

With the consent of the patient’s family, samples from the child and family members were collected to conduct whole-exome sequencing of potential genetic diseases. Due to the child’s fungal infection and immune deficiency, whole-exome sequencing results were analyzed and sorted using Exomiser Software, and *CARD9* and *CD40LG* mutations were screened. A hemizygous missense variant, c.346G > A (p.Gly116Ser), was detected on the patient’s *CD40LG* gene (**Figure 4**) that was not present in either parent’s validation samples. The activated T cells from the patient had 52% CD40L expression as compared to 80.7% expression in a healthy control (**Supplementary Figure S1**). This finding was supported by abnormal immunoglobulin levels. A heterozygous frameshift variant, c.820dup (p.Asp274GlyfsTer61), was also detected in *CARD9* and found to be inherited from the patient’s mother (**Figure 4**). The 3D structure of the patient’s CARD9 protein differed from the wild-type protein (**Supplementary Figure S2**). The patient’s family history revealed that all immediate family members were healthy except for the child who had a history of poor immune function. Notably, the child and his two brothers were born prematurely at approximately 33 weeks of gestation. His mother reported that she suffered from mild dermatitis, including tinea pedis, and had generally self-medicated for this condition.

After TM infection was suggested by peripheral blood smear, voriconazole and amphotericin B treatment was administered, followed by chemotherapy for HLH. On the fourth day of administration, the abnormal cell clusters in the patient’s blood routine scatter diagram disappeared (**Supplementary Figure S3**). During treatment, the child had a series of complications caused by severe sepsis, including multiple organ dysfunction syndrome and fungal enterocolitis complicated with massive bleeding. However, due to the early implementation of antimicrobial and symptomatic treatment, the patient was finally cured and discharged after 2 months.

## Discussion

TM is the only temperature biphasic opportunistic fungus in the *Penicillium* genus. At 25°C, TM exhibits a hyphal phase, while at 37°C, TM has a yeast-like morphology and exhibits pathogenicity. This fungus often has microscopically divided oval yeast cells and is associated with primary immunodeficiency, malignant tumors, organ transplantation, autoimmune diseases, and the application of immune agents (11). TM primarily invades monocytes and macrophages and often disseminates throughout the body. Due to the lack of specific clinical manifestations, TM infection is easily misdiagnosed.

Culture is used as the gold standard for laboratory diagnosis; however, the 7–10-day culture period can delay lifesaving treatment, and the long time period needed for early definitive diagnosis is a primary reason for the high mortality rate associated with TM (5). Both mNGS and PCR are rapid and reliable but require expensive laboratory equipment and cannot be performed in remote areas (12). Microscopy can offer a quick diagnosis but is dependent on the experience of the microscopist and is prone to misdetections and misdiagnoses. Human resistance to TM is dominated by Th1-mediated cellular immunity, a process that involves phagocytosis by macrophages and delayed hypersensitivity mediated by sensitized T cells (13). The cytomorphological analyses of the case described here revealed abnormally phagocytosed mature granulocytes (**Figure 1**), which showed aggregated or scattered fungal spores both inside and outside the cells. The fungal cells were oval-shaped, and some contained a transverse septum which is a marker of their division and the most characteristic manifestation of TM. Fungal spores were also found in subsequent bone marrow smears. The disease should be considered in patients with prolonged fever, respiratory symptoms, hepatosplenomegaly or lymph node enlargement, anemia, fungal rash, residence in a TM endemic area or who recently returned from travel to an endemic area, or in workers performing TM-related experiments.

HLH is a multi-organ excessive inflammatory syndrome caused by genetic or acquired abnormalities in immune regulation (14). In the case described here, HLH-associated genes, such as *FSH-1*, *RAB27-A*, and *SH2D1A*, and hemophagocytic protein levels revealed no abnormalities, so primary HLH was excluded. The blood culture and mNGS results suggested TM infection, and there was no evidence of autoantibodies or tumors, no history of organ or hematopoietic stem cell transplantation, and no HLH-related metabolic diseases and drug administration. Thus, TM-associated secondary HLH was considered. HLH is a rapidly progressing and highly fatal disease in children (1), and the possibility should also be carefully considered if there are two or three lines of peripheral blood reduction that require the identification of leukemia.

HIV-negative children infected with TM have primary immunodeficiency diseases (PID) (15–17), such as *STAT1* mutations, that result in enhanced IFN-α/β signaling, lower INF-γ, IL-17, and IL-22 production, and defective Th1/Th17 responses. The development of next-generation sequencing technology has increased the concern about children with PID. In the current study, whole-exome sequencing revealed two mutations associated with the case’s clinical phenotype. This included a mutation in exon 3 of the *CD40LG* gene, c.346G > A p.Gly116Ser. This mutation, which results in little to no expression of CD40L on the surface of T cells, is most likely to cause HIGM (18). If CD40L is unavailable to stimulate CD40, the immunoglobulin secreted by B cells has a class conversion disorder and affects the costimulatory ability of T cells (6, 19). CD40 is also important for the activation of phagocytes, including monocytes and dendritic cells, putting these patients at a higher risk for opportunistic infections such as that of *Pneumocystis carinii* and *Cryptosporidium* spp. In clinical practice, CD40L deficiency is associated with combined immunodeficiency and increased susceptibility to fungal infections (20). In southeast Asia and southern China, patients with CD40L deficiency are particularly susceptible to TM (21). In the current study, flow cytometry revealed a reduced CD40L expression on the patient’s activated T cells. While most *CD40LG* mutations result in a lack of protein expression, there are cases in which CD40L protein expression is normal or reduced. These findings highlight the importance of conducting CD40L gene sequencing for patients with normal CD40L protein expression who have clinical manifestations consistent with HIGM (22–24). Researchers have suggested using immunoglobulin profiles and cellular immune function tests to aid in HIGM diagnoses (25). It may also be worthwhile testing for memory B cells, which are significantly lower in HIGM patients (26). A retrospective analysis of the case’s immunoglobulin results before the first admission and after several follow-up treatments revealed that while the IgM levels were normal, the IgG and IgA levels were low, a finding consistent with his immunocompromised status and reduced CD40L expression. This deficiency, in most isotypic immunoglobulins, makes the affected individuals highly vulnerable to recurrent bacterial infections (8, 27).

The current case also had repeated infections and was hospitalized several times for pneumonia and inflammatory bowel disease within 6 months after being cured of TM. Thus, it is probable that the child had primary immunodeficiency with HIGM. No *CD40LG* relevant mutations were detected in the paternal validation samples; however, a heterozygous frameshift variant, c.820dup (p.Asp274GlyfsTer61), on exon 6 of the *CARD9* gene, was found to be inherited from the mother. CARD9, a member of the CARD family, is an important connexin discovered by Bertin et al. (28), which effectively integrates recognition signals from various natural immune receptors and plays an important role in antifungal immunity. The c.820dup homozygous variant and the compound heterozygous variant, c.820dup/c.68C > A, have been detected in multiple patients with fungal infections (29–31). Most individuals with heterozygous CARD9 mutations, including the case’s mother, are healthy carriers and are not impacted by severe fungal infections. However, using Exomiser Software analysis and protein tertiary structure prediction, the c.820dup/c.68C > A variant is associated with an increased risk of infection. Due to the rare constraints of the clinical samples, it was not determined whether the c.820dup variant affected the CARD9 protein expression and function and will thus require further in-depth study.

The current case study described a child with PID, a risk factor for TM infection. In PID patients, macrophages that have engulfed fungi cannot be activated and sterilized, triggering uncontrolled macrophage proliferation. This case was also initially misdiagnosed as pulmonary tuberculosis, delaying the treatment by a week. When intracellular fungi are permitted to multiply, bacteria-containing macrophages migrate through the lymph and blood, causing a disseminated systemic infection. If a significant macrophage proliferative response occurs in the systemic reticuloendothelial system, HLH mechanisms become triggered. Early and effective antifungal therapy is the most important way to improve the prognosis. A retrospective analysis of non-HIV-infected TM children in southern China indicated that intravenous voriconazole is the initial antifungal agent used in children (32). A randomized controlled study of anti-TM drugs in Vietnam found that amphotericin B treatment was associated with better clinical efficacy, higher fungal clearance, and significantly reduced recurrence than itraconazole among HIV-infected individuals (5). Previous cases of patients with CD40L deficiency or those facing life-threatening potential infections, including a case of TM, have died of multi-organ failure (19). The case described in the current study had HLH and developed multi-organ failure, combined with pneumonia, fungal enterocolitis, and other serious complications, but survived thanks to the early detection of TM and effective antifungal therapy.

In the case study presented here, a patient was found with an abnormal cell mass classification that was later identified as containing fungus-phagocytic mature granulocytes. The case was initially suspected of having a TM infection that was used to guide the follow-up clinical examinations and treatments. This case study should be used to inform laboratory morphological testing. The findings illustrate that infection-associated secondary HLH, especially among HIV-negative individuals infected with TM, should be evaluated for immunodeficiency.

## Data availability statement

The original contributions presented in the study are included in the article/**Supplementary Material**. Further inquiries can be directed to the corresponding author.

## Ethics statement

Written informed consent was obtained from the individual(s), and minor(s)’ legal guardian/next of kin, for the publication of any potentially identifiable images or data included in this article.

## Author contributions

All authors listed have made a substantial, direct, and intellectual contribution to the work, and approved it for publication.

## Funding

This work was supported by Guangdong Basic and Applied Basic Research Foundation (2021A1515220072) and Guangdong High-Level Hospital Construction Fund. Shenzhen Key Medical Discipline Construction Fund (SZXK054).

## Acknowledgments

The authors would like to thank Dr. Wujiao Li and Dr. Zhihao Xing from the Clinical Laboratory of Shenzhen Children’s Hospital for providing bioinformatics consultation on this paper.

## Conflict of interest

The authors declare that the research was conducted in the absence of any commercial or financial relationships that could be construed as a potential conflict of interest.

## Publisher’s note

All claims expressed in this article are solely those of the authors and do not necessarily represent those of their affiliated organizations, or those of the publisher, the editors and the reviewers. Any product that may be evaluated in this article, or claim that may be made by its manufacturer, is not guaranteed or endorsed by the publisher.
