# Supplementary material for: Case report: Hemophagocytic lymphohistiocytosis in a child with primary immunodeficiency infected with Talaromyces marneffei
Source: Front Immunol. 2022 Dec 2;13:1038354. doi: 10.3389/fimmu.2022.1038354 (PMC9755863; doi:10.3389/fimmu.2022.1038354)
Supplement: Supplementary file 1 [file DataSheet_1.pdf]

Hemophagocytic lymphohistiocytosis in a child with primary immunodeficiency infected with *Talaromyces marneffei*

Supplementary materials

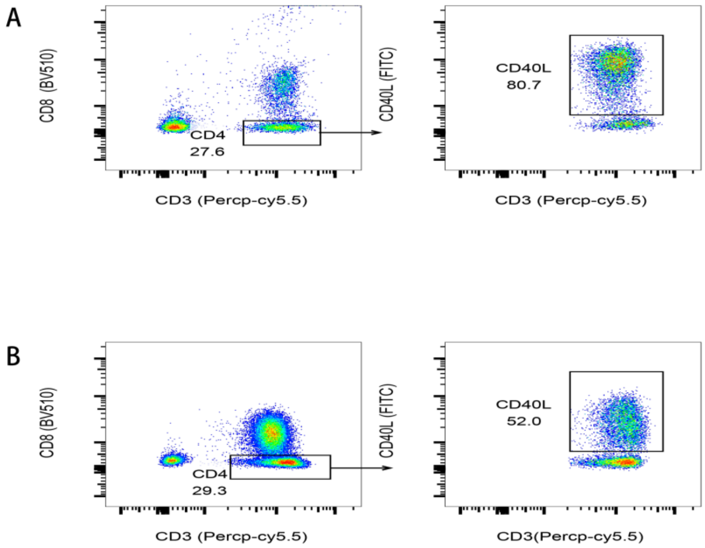

Supplementary Figure 1.CD40L expression on the surface of activated Th cells. (A) CD40L expression in a healthy control as detected by flow cytometry. (B) CD40L expression in the case with the CD40*LG* mutation as detected by flow cytometry.

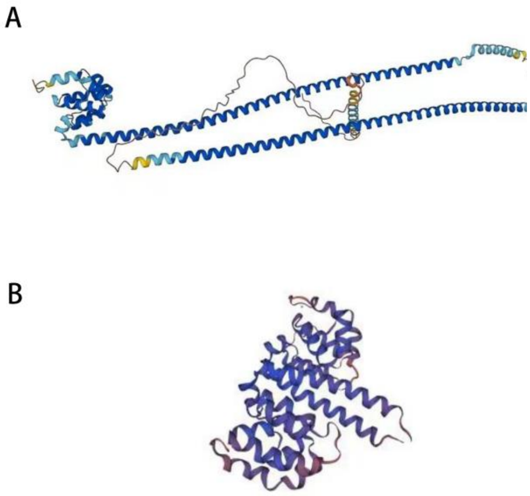

Supplementary Figure 2. Structural analysis of the *CARD9* mutation. (A) The *CARD9* wild-type three-dimensional structure was predicted by Alphafold and downloaded from its protein structure database. (B) The three-dimensional structure of the *CARD 9* mutant was predicted using the Swiss-Model.

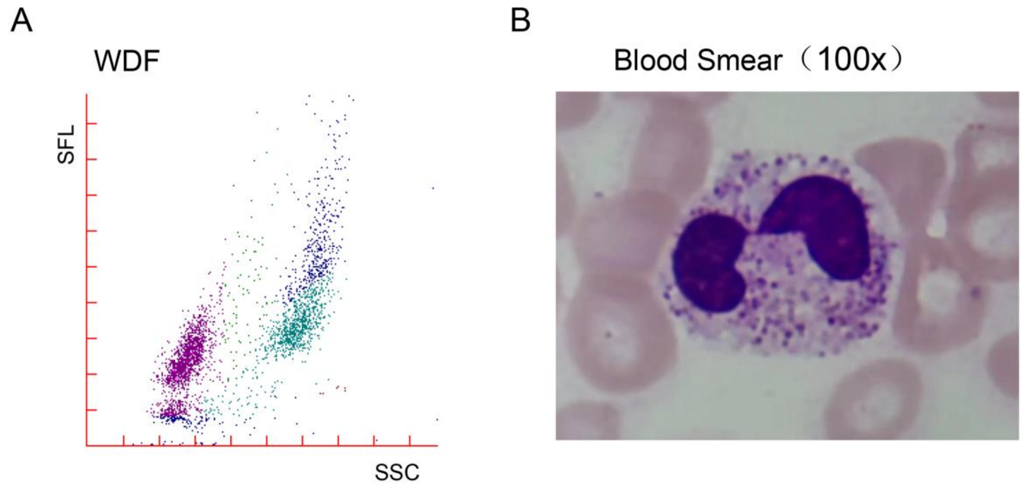

Supplementary Figure 3. Post-treatment white blood cell count analysis and microscopic morphology. (A) After antifungal treatment, the WDF showed correct differential WBCs and mature granulocytes were divided into green areas. (B) Microscopic examination of peripheral blood smears did not reveal fungal spores or phagocytosis of fungi in mature granulocytes.

Supplementary Table 1. Results of the patient's immunoglobulin level before each treatment

Date（2021–2022）	IgG (5.28–21.90g/L)	IgM (0.48–2.26g/L)	IgA (0.41–2.97g/L)
October 2nd	3.85	1.80	0.33
December 4th	4.46	1.06	0.05
February 9th	3.42	2.17	0.12
June 3rd	2.92	3.14	0.03
